# Molecular Characterization of Viruses Revealed by Japanese Flowering Cherry (*Prunus serrulata*) Virome Analysis

**DOI:** 10.3390/ijms27125478

**Published:** 2026-06-17

**Authors:** Anna Sheveleva, Fedor Sharko, Elena Motsar, Natalia Karpun, Sergei Chirkov

**Affiliations:** 1 Virology Department, Lomonosov Moscow State University, Moscow 119234, Russia; anncsh@yandex.ru (A.S.); elena.motsar31@gmail.com (E.M.); 2 National Research Center “Kurchatov Institute”, Moscow 123182, Russia; fedosic@gmail.com; 3 Federal Research Centre the Subtropical Scientific Centre of the Russian Academy of Sciences, Sochi 354002, Russia; nkolem@mail.ru

**Keywords:** Japanese flowering cherry, virome, cherry virus A, little cherry virus 2, American plum line pattern virus, prune dwarf virus, alfalfa mosaic virus, high-throughput sequencing

## Abstract

Japanese flowering cherry (*Prunus serrulata* Lindl.) is a deciduous tree with great decorative value due to its splendid spring bloom. Flowering cherry is native to East Asia and is cultivated in many other parts of the world. Two trees with virus-like symptoms on leaves, denoted by S14 and S27, were found in the town of Sochi, Russia. Virome analysis of these trees using high-throughput sequencing revealed the presence of several viruses, including little cherry virus 2 (LChV-2), American plum line pattern virus (APLPV), and cherry virus A (CVA) in the S14 tree, as well as CVA, prune dwarf virus (PDV), and alfalfa mosaic virus (AMV) in the S27 tree. Nearly complete genomes of the detected viruses have been recovered and characterized. In the S14 tree, CVA was represented by three diverse genotypes sharing 80.8–83.5% nt identity. The complete APLPV genome from flowering cherry has been first sequenced. AMV was detected on *Prunus* species for the first time. This is the first report of LChV-2 from Russia. Thus, PDV, APLPV, AMV, LChV-2, and CVA were first found on *P. serrulata* from Russia, adding to the information on their genetic diversity, host range, and global geographical distribution.

## 1. Introduction

Japanese flowering cherry (genus *Prunus* in the family *Rosaceae*) is native to Eastern Asia and is one of the most famous species of ornamental woody plants, valued for its lush spring blooming. A group of close species, including *P. serrulata* Lindl., *P. jamasakura* (Siebold) Koidz., and *P. leveilleana* Koehne with several related infraspecific taxa, are collectively considered *P. serrulata*. This species is widely cultivated in Japan and other East Asian countries, and many other parts of the world, for garden arrangements and landscape design. The *P. serrulata* garden cherry is currently represented by over two hundred forms, with double flowers ranging from white to bright pink shades, among which the cultivars (cv.) Kanzan, Shirofugen, and Amanogawa are most known, as well as a few others [1,2].

Over a dozen viruses from different taxonomic groups have been reported in flowering cherry to date [3,4,5,6,7,8]. These include apple mosaic virus, prune dwarf virus (PDV), prunus necrotic ringspot virus [4,6] and American plum line pattern virus (APLPV) [9,10] (all four are from the genus *Ilarvirus*, family *Bromoviridae*); cherry green ring mottle virus and cherry necrotic rusty mottle virus (genus *Robigovirus*, *Betaflexiviridae*) [11,12]; cherry virus A (CVA) (genus *Capillovirus*, *Betaflexiviridae*) [7,13]; little cherry virus 1 (genus *Velarivirus*, *Closteroviridae*) [14,15]; little cherry virus 2 (LChV-2) and plum bark necrosis stem pitting-associated virus (genus *Ampelovirus*, *Closteroviridae*) [16,17,18]; strawberry latent ringspot virus (genus *Sadwavirus*, *Secoviridae*) [4]; and plum pox virus (genus *Potyvirus*, *Potyviridae*) [19]. In addition, the coat protein (CP) gene sequences of cherry virus T (genus *Robigovirus*, *Betaflexiviridae*) from *P. serrulata* were deposited in GenBank under accession numbers PQ422079-PQ422080 (unpublished).

Two *P. serrulata* trees with virus-like symptoms on leaves were found during an urban green plantings survey in the town of Sochi. This town is located on the Black Sea coast of the Caucasus in Russia. Virome analysis of these trees using high-throughput sequencing (HTS) has revealed the presence of PDV, APLPV, LChV-2, CVA, as well as alfalfa mosaic virus (AMV, genus *Alfamovirus*, *Bromoviridae*).

Typical of the family *Bromoviridae*, PDV, APLPV, and AMV have a segmented genome consisting of three single-stranded, positive-sense RNAs, each packed individually into isometric (PDV, APLPV) or bacilliform (AMV) virus particles. The isometric particles are 26–36 nm in diameter. The bacilliform particles are 19 nm wide and have lengths of 56 nm, 43 nm, 35 nm, and 30 nm depending on the encapsidated RNA size. The 5′ end of genomic RNAs is capped, and the 3′ terminal sequence is folded in a tRNA-like structure, which converts into the CP binding site while the ilarvirus or AMV genome is activated. The activation is initiated by the binding of the CP to the ATGC sequences situated at the 3′ end of genomic RNAs. Four open reading frames (ORFs) encode replicase (ORF1), which includes methyltransferase (MTR) and helicase (HEL) domains, RNA-dependent RNA polymerase (RdRp, ORF2), movement proteins (MP, ORF3a), and CP (ORF3b). Unlike most ilarviruses, RNAs 2 of PDV, APLPV, and AMV are monocistronic. They lack ORF2b, which encodes a gene silencing suppressor required for the systemic transport of these viruses. Under natural conditions, PDV and APLPV infect only *Prunus* spp. In contrast, AMV has a very wide host range, but it has not yet been described on flowering cherry or *Prunus* species in general [20,21,22,23].

CVA is one of the most common *Prunus* viruses that occurs in sweet cherry (*P. avium*) and sour cherry (*P. cerasus*) worldwide and was also detected on peach (*P. persica*), apricot (*P. armeniaca*), plum (*P. domestica*), myrobalan (*P. cerasifera*), Japanese plum (*P. salicina*), Japanese apricot (*P. mume*), and Japanese flowering cherry (*P. serrulata*). CVA virions are flexuous filaments 640–700 nm in length. The genome is single-stranded positive-sense polyadenylated RNA of about 7.5 kb with two ORFs. ORF1 is flanked by the 5′ and 3′ untranslated regions (UTRs) and encodes replication-associated proteins and the CP. ORF2 is nested within ORF1 in a different reading frame and encodes the MP [24,25,26].

LChV-2 belongs to subgroup I of the genus *Ampelovirus* in the family *Closteroviridae*. Ampeloviruses have filamentous virions 1400–2200 nm long and 12 nm in diameter and vary widely in genome size and organization. The LChV-2 genome is single-stranded RNA of about 15 kb, which usually contains ten ORFs encodi,ion-associated proteins (MTR, HEL, RdRp), two types of CPs (the genuine CP and a CP duplicate, or CP minor (CPm)), the heat shock HSP70 and HSP90 homolog proteins, a small hydrophobic p6 protein, as well as the p22 and p26 proteins of unknown function. The LChV-2 genome is potentially ambisense, with a negative-sense ORF0 at the 5′ end of genomic RNA, from which an 18K protein of unknown function can be translated in vitro. The CP, CPm, HSP70, HSP90, and p6 form a five-gene module that is required for virus cell-to-cell movement. As with the majority of closteroviruses, LChV-2 is a phloem-limited virus. Its host range seems to be limited to the *Prunus* species. LChV-2 is the primary etiological agent of the little cherry disease, which causes a significant reduction in the yield and quality of sweet and sour cherry fruits. At the same time, ornamental *Prunus* spp. can be latently infected with this virus [27,28].

The objectives of this study were to complete genome sequencing and molecular characterization of viruses found on flowering cherry trees from the Black Sea coast of the Caucasus in Russia.

## 2. Results

### 2.1. Identification of Viruses from P. serrulata Trees

**Figure 1:**
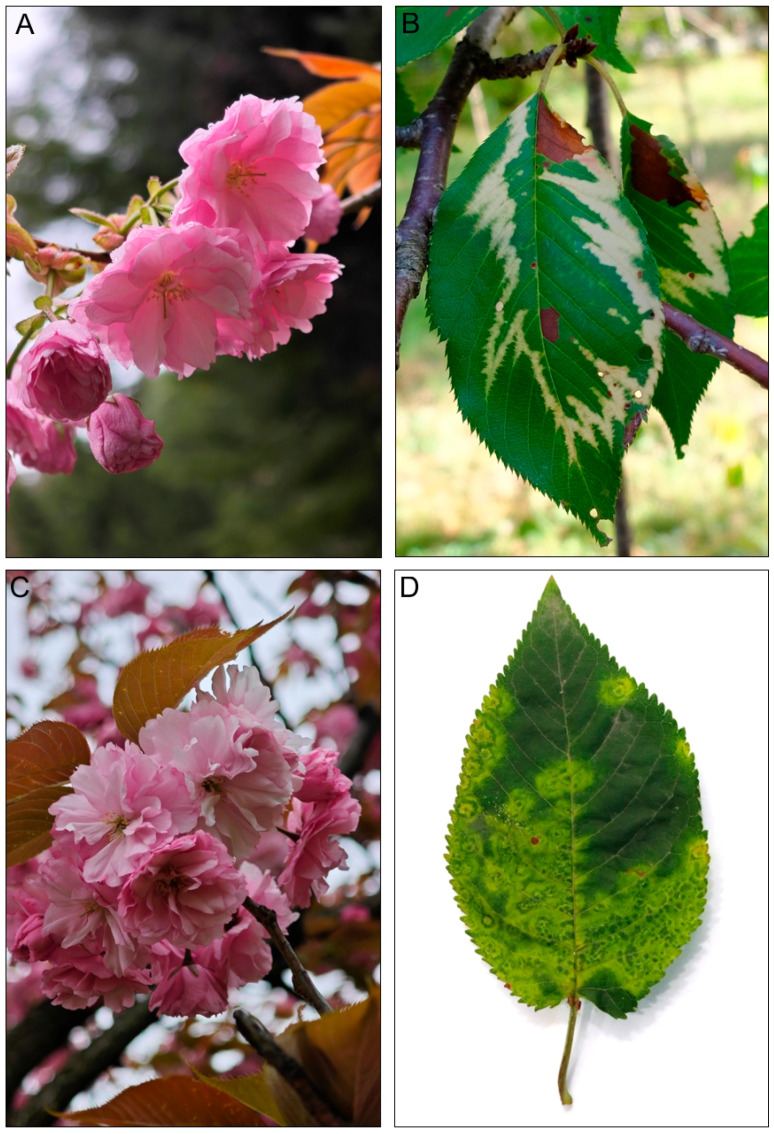
Flowers and symptomatic leaves from *Prunus serrulata* cultivar Kiku-Shidare (**A**,**B**), tree S14, and *P. serrulata* cultivar Kanzan (**C**,**D**), tree S27. Leaves were collected in June 2024. The cherry blossom photographs were taken in April 2026.

Two flowering cherry trees with virus-like foliar symptoms on leaves were found in the urban green plantings in the town of Sochi (Figure 1). The first tree was *P. serrulata* cv. Kiku-Shidare and the second one was *P. serrulata* tree cv. Kanzan. These specimens were denoted by S14 and S27, respectively. Results of the virome analysis of the symptomatic leaves from both trees are presented in Table 1. The average depth of coverage, calculated by mapping reads to the assembled viral genomes, was used as a proxy for the relative abundance of each viral RNA in the samples.

**Figure 2:**
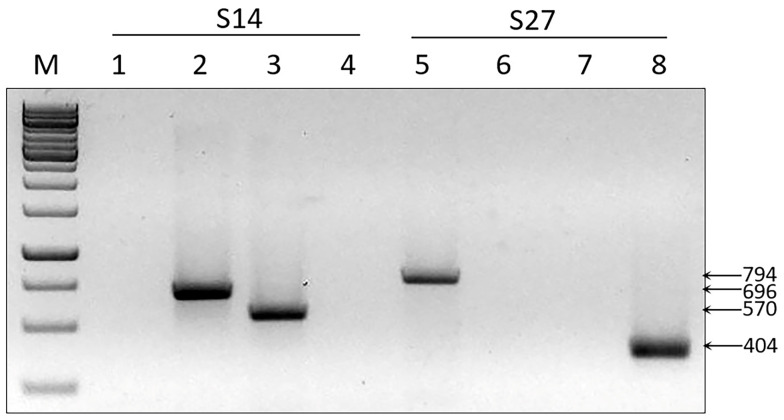
Agarose gel electrophoresis of amplicons obtained by RT-PCR assay of the S14 and S27 samples using virus-specific primers to prune dwarf virus (lines 1, 5), little cherry virus 2 (lines 2, 6), American plum line pattern virus (lines 3, 7), and alfalfa mosaic virus (lines 4, 8). M—GeneRuler 1 kb DNA ladder (Thermo Scientific, Waltham, MA, USA). The arrows to the right of the figure indicate PCR products of corresponding sizes (bp).

**Figure 3:**
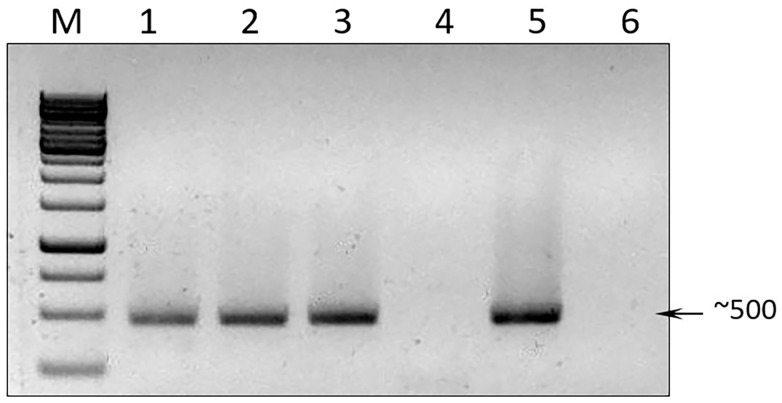
Agarose gel electrophoresis of amplicons obtained by RT-PCR assay of the S14 (lanes 1–3) and S27 (lanes 4–6) samples using primers specific to different cherry virus A (CVA) isolates: CVA-S14-84 (lanes 1,4), CVA-S14-85 (lanes 2,5), and CVA-S14-86 (lanes 3,6). M—GeneRuler 1 kb DNA ladder (Thermo Scientific, Waltham, MA, USA). The arrow to the right of the figure indicates the PCR product of the corresponding size (bp).

Contigs corresponding to nearly complete genomes of APLPV, CVA, and LChV-2, as well as PDV, CVA, and AMV, were assembled in the S14 and S27 samples, respectively. All the detected viruses were confirmed by conventional RT-PCR (Figure 2 and Figure 3) using virus-specific primers (Table S1) and were validated by direct sequencing of PCR products. 

### 2.2. Molecular Characterization of Detected Viruses

#### 2.2.1. Prune Dwarf Virus

Five PDV-related contigs, ranging from 1011 nt to 2661 nt in length, were obtained from the S27 sample. A nearly complete PDV genome was assembled from these contigs. RNA1 of the PDV-S27 isolate 3354 nt length contained ORF1 of 3168 nt encoding the 119K replicase of 1055 amino acid (aa) residues. The viral MTR (pfam01660) and viral RNA HEL (pfam01443) motifs were predicted at aa positions 95–394 and 766–1025, respectively. RNA2 2577 nt long contained ORF2 of 2364 nt encoding the 89K Bromoviridae_RdRp (cd23252) of 787 aa. ORF3a and ORF3b of 882 nt and 657 nt, encoding the 32K Bromo_MP (pfam01573) and 24K Ilar_coat CP (pfam01787) of 293 aa and 218 aa, respectively, were found in RNA3 of 2110 nt length. ORF3a and ORF3b were separated by the intergenic region of 72 nt. The 3′ end of genomic RNAs and the N-terminus of CP were found to contain a series of ATGC sequences and a key arginine (R) residue within the ^3^GKATKSGKPTTRSQSFALARK2^3^ domain, which are both involved in the viral genome activation [20,21].

RNA1 of the PDV-S27 isolate was the closest to the Russian PDV isolate from plum (OQ995020) (95.0% identity), while RNA2 and RNA3 were most closely related (93.7% identity) to the Canadian PDV isolates from cherry (MZ220878 and MZ220998, respectively). On the other hand, the only complete PDV genome from flowering cherry currently available in GenBank is the Canadian isolate 13C206 (accession numbers MZ220974, MZ220865, MZ220994) [13]. The PDV-S27 and 13C206 isolates were distantly related to one another, sharing 91.8%, 86.1%, and 97.7% identity for RNA1, RNA2, and RNA3, respectively.

**Figure 4:**
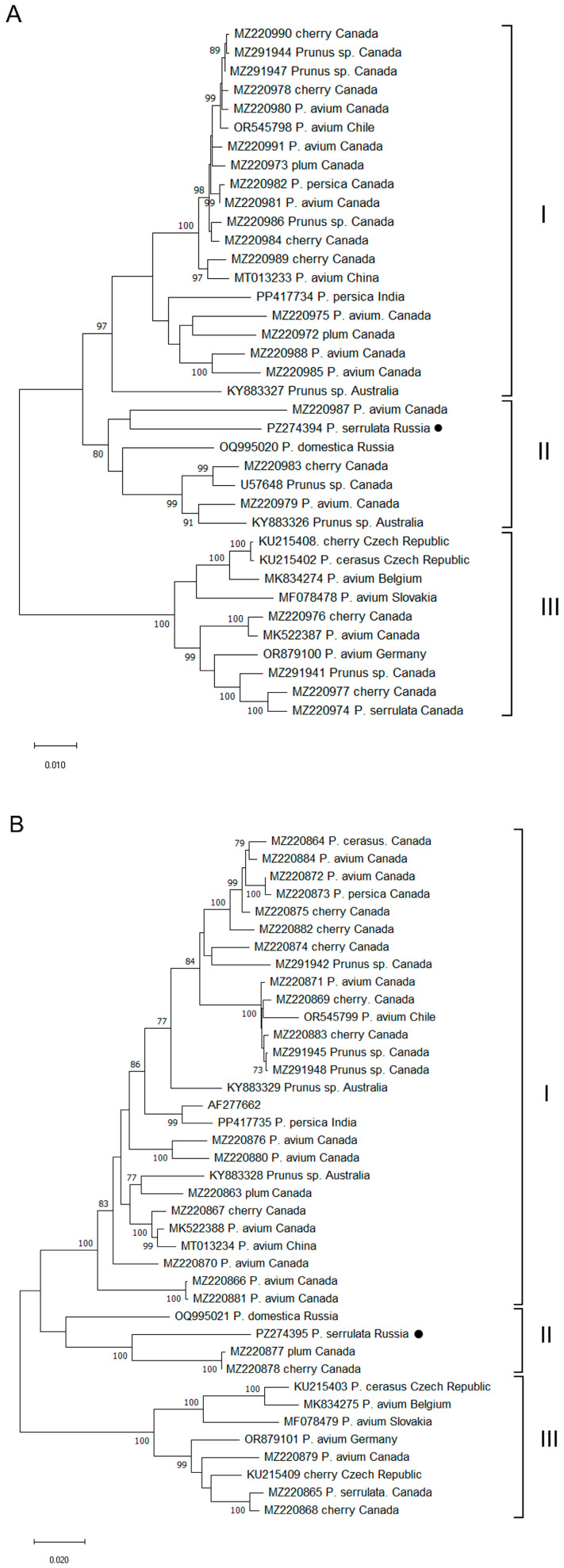

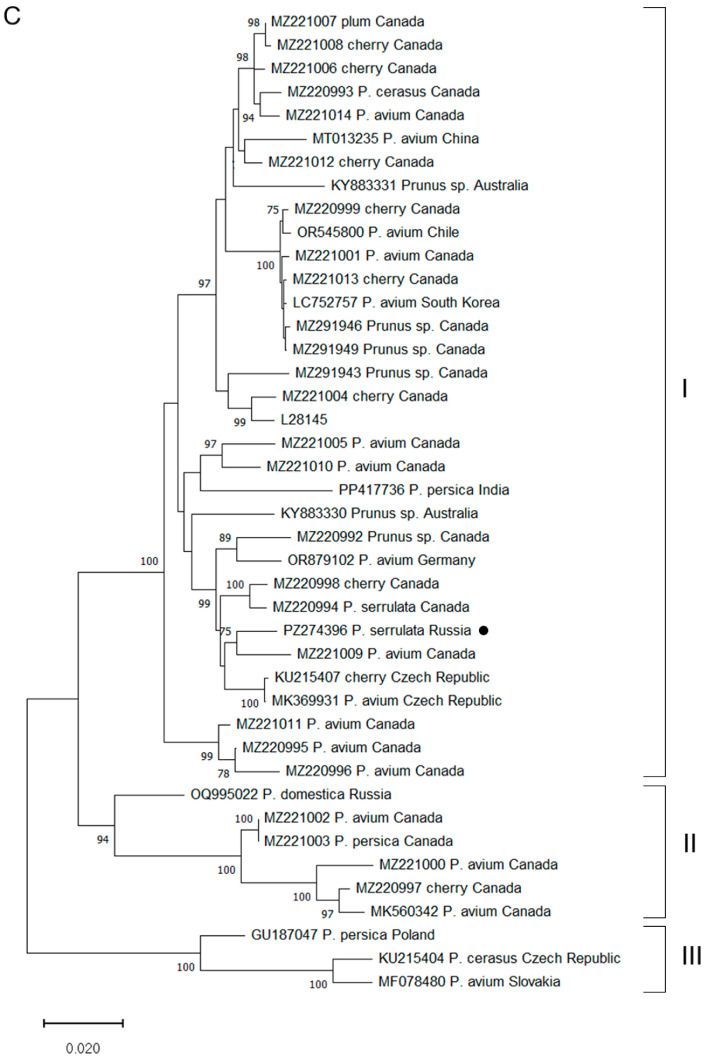
Maximum likelihood phylogenetic trees based on full-length sequences of prune dwarf virus (PDV) genome RNA1 (**A**), RNA2 (**B**), and RNA3 (**C**) using the GTR best-fit substitution model. The accession number, Latin name of the host plant, and geographic origin of the virus isolate are given at the end of the branches. Russian PDV isolate from *Prunus serrulata* is marked with a black circle (•). Brackets combine isolates belonging to clusters I, II, or III. Bootstrap values from 1000 replicates (>75%) are shown next to the corresponding node. The scale bar indicates the number of nucleotide substitutions per site.

Three main clusters were formed in the phylogenetic analysis of PDV genome RNAs. Consistent with their nt sequence identity values, RNA1 and RNA2 of both PDV isolates from flowering cherry were shown to group into different clusters, while RNAs 3 were assigned into the same cluster (Figure 4). No clear phylogenetic grouping based on the host or the geographical origin was observed for PDV genome RNAs.

The nearly complete genome sequences of the Russian PDV isolate from flowering cherry were deposited in GenBank under accession numbers PZ274394-PZ274396. This is the first report of PDV from flowering cherry in Russia, expanding the information on its geographic distribution and genetic diversity.

#### 2.2.2. American Plum Line Pattern Virus

Five APLPV-related contigs ranging from 549 nt to 3358 nt in length were obtained in the S14 sample and a nearly complete APLPV genome was assembled. RNA1 of the APLPV-S14 isolate 3359 nt in length contained ORF1 of 3198 nt encoding the 121K replicase of 1065 aa. The viral MTR (pfam01660) and viral RNA HEL (pfam01443) motifs were predicted at aa positions 93–388 and 766–1026, respectively. RNA2 of 2379 nt contained ORF2 2091 nt long encoding the 80K Bromoviridae_RdRp (cd23252) of 696 aa. RNA3 2030 nt in length included ORF3a and ORF3b of 810 nt and 654 nt, encoding the 31K Bromo_MP (pfam01573) and 24K Ilar_coat CP (pfam01787) of 269 aa and 217 aa, respectively. ORF3a and ORF3b were separated by the intergenic region of 241 nt. As with PDV, a series of ATGC sequences was presented at the 3′ end of genomic RNAs. The putative critical arginine (R) residue was found to be conserved within a ^15^QNARTTQFAQR**R**AAAARAEIE^35^ domain at the N-terminus of the CP [20]. Nearly complete RNA1, RNA2, and RNA3 sequences of the APLPV-S14 isolate were the closest to the Indian PM1 isolate from *P. domestica* cv. Stanley (OQ513268-OQ513270) with 98.3–98.9% nt identity. Furthermore, corresponding regions of the APLPV-S14 genome were 97.7–99.2% identical to partial (343–904 nt long) genome sequences of the Japanese (KY883666-KY883669) [10] and Italian (OP972867-OP972868) [9] APLPV isolates from *P. serrulata*.

**Figure 5:**
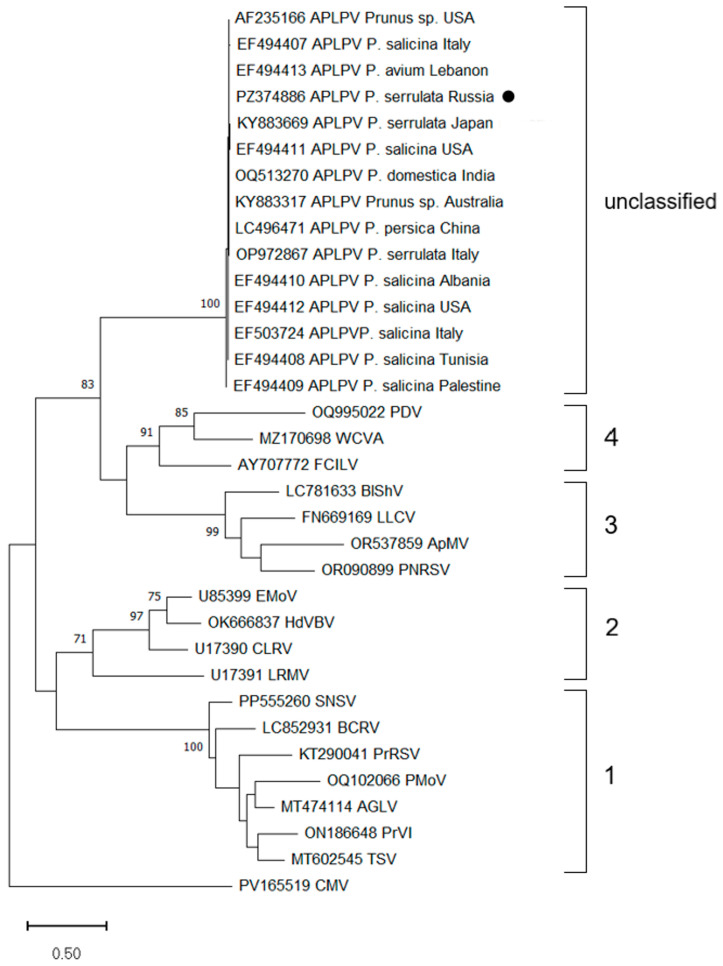
Phylogenetic analysis of American plum line pattern virus (APLPV) isolate from *P. serrulata* and selected members of the genus *Ilarvirus* based on the alignment of the complete amino acid sequences of the coat protein. The tree was reconstructed in MEGA X using the maximum likelihood method with the WAG best-fit substitution model. Bootstrap values (>70%) from 1000 replicates are shown next to the corresponding nodes. The accession numbers in GenBank and virus acronyms are shown at the end of branches. Brackets combine isolates of five ilarvirus subgroups. The scale bar indicates the number of substitutions per nucleotide. The Russian APLPV isolate is highlighted with a black circle (•). Viruses used for tree reconstruction are (subgroup 1) AGLV—ageratum latent virus, BCRV—blackberry chlorotic ringspot virus, PMoV—parietaria mottle virus, PrRSV—privet ringspot virus, PrVI—prunus virus I, SNSV—strawberry necrotic shock virus, and TSV—tobacco streak virus; (subgroup 2) CLRV—citrus leaf rugose virus, EMoV—elm mottle virus, HdVBV—hydrangea vein banding virus, LRMV—lilac ring mottle virus; (subgroup 3) ApMV—apple mosaic virus, BlShV—blueberry shock virus, LLCV—lilac leaf chlorosis virus, PNRSV—prunus necrotic ringspot virus; (subgroup 4) FClLV—fragaria chiloensis latent virus, PDV—prune dwarf virus, WCVA—water chestnut virus A; (unclassified) APLPV—American plum line pattern virus. Cucumber mosaic virus (CMV) was used as a phylogenetic outgroup.

Phylogenetic analysis of the aa sequences of ilarvirus CPs available in GenBank showed their grouping into five clusters, typical of ilarviruses. The Russian APLPV isolate was assigned to a cluster formed by isolates of this virus from different hosts and geographic locations (Figure 5). The APLPV isolates from this cluster were 98.1–99.9% and 98.1–100% identical at the nt and aa levels, respectively, indicating very low genetic variability of APLPV isolates of different origins. The remarkable exception was the divergent Japanese isolate Nar from *P. serrulata* (KY883669) [10], which was 89.2–90.2% and 89.8–91.4% identical to the rest of the isolates from this cluster at the nt and aa levels, respectively. The high level of the CP identity irrespective of the APLPV origin can indicate genetic stability of this virus.

The nearly complete genome sequences of the APLPV flowering cherry isolate were deposited in GenBank under accession numbers PZ374884-PZ374886. This is the first complete APLPV genome from *P. serrulata* and the first report of APLPV from Russia, adding information on its geographic distribution.

#### 2.2.3. Alfalfa Mosaic Virus

A nearly complete AMV genome was assembled from three AMV-related contigs, 3630 nt, 2559 nt, and 2011 nt in length, obtained from the S27 specimen. RNA1 of the AMV-S27 isolate contained ORF1 of 3381 nt encoding 126K replicase of 1126 aa. The MTR and HEL motifs were predicted at aa positions 68–404 and 834–1094, respectively. RNA2 contained ORF2 2373 nt long encoding 90K Bromoviridae_RdRp (cd23252) of 790 aa. ORF3a and ORF3b of 903 nt and 666 nt, encoding 32K Bromo_MP (pfam01573) and 24K Ilar_coat CP (pfam01787) of 300 aa and 221 aa, respectively, were found in RNA3. ORF3a and ORF3b were separated by the intergenic region of 50 nt. Both ATGC motifs and the conserved arginine residue at position 18 involved in genome activation [20,22] were identified at the 3′ end of genomic RNAs and at the N-terminus of CP, respectively. RNA1 and RNA3 were most closely related to the German PV-1411 isolate from *Origanum vulgare* (OR607775, OR607777) (98.7% and 97.2% identity, respectively), while RNA2 was the closest (97.3% identity) to the Italian See-1 isolate from *Sechium edule* (MT093210). The sequences of AMV genomic RNAs 1, 2, and 3 were deposited in GenBank under accession numbers PZ374887-PZ374889.

**Figure 6:**
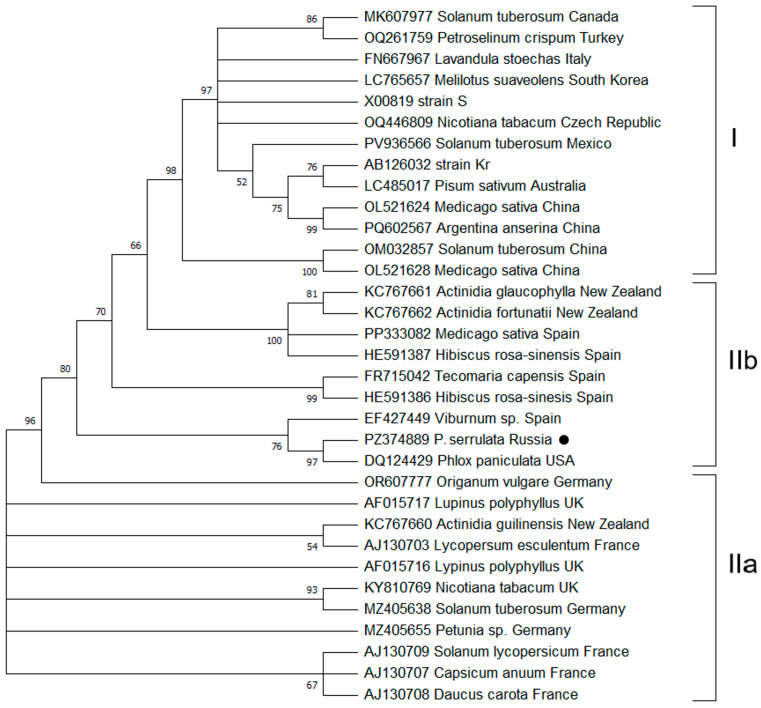
Phylogenetic analysis of alfalfa mosaic virus (AMV) isolate from *P. serrulata* and selected AMV isolates available from GenBank based on the alignment of the complete nucleotide sequences of the coat protein gene. The bootstrap consensus tree was reconstructed in MEGA X using the maximum likelihood method with the GTR best-fit substitution model. The tree inferred from 1000 replicates was used to reconstruct the evolutionary history of the taxa analyzed. Branches corresponding to partitions reproduced in less than 50% of bootstrap replicates were collapsed. The percentage of replicate trees in which the associated taxa clustered together in the bootstrap test (1000 replicates) is shown next to the branches. The accession number, Latin name of the host plant, and geographical origin of the virus isolate are given at the end of the branches. Brackets combine isolates belonging to clusters I, IIa, and IIb. The scale bar indicates the number of nucleotide substitutions per site. Russian *P. serrulata* isolate is marked with a black circle (•).

Based on phylogenetic analysis of the CP gene, known AMV isolates can be divided into three groups, I, IIA, and IIB, according to their geographical origin [29]. The clade IIB included the Spain AMV isolates from Cape honeysuckle, hibiscus rose-sinensis, viburnum, alfalfa, as well as the New Zealand isolates from actinidia and the American isolate from phlox. The Russian AMV isolate from *P. serrulata* was also assigned to group IIB, thus expanding this clade and increasing the AMV genetic diversity and geographical distribution (Figure 6).

To the best of our knowledge, this is the first report on AMV infection in *P. serrulata* and *Prunus* spp. in general.

#### 2.2.4. Little Cherry Virus 2

Six LChV-2-related contigs from 1589 nt to 10,185 nt in length were obtained in the S14 sample. The nearly complete LChV-2 genome of 15,048 nt was assembled from these contigs. Ten ORFs have been predicted in the newly sequenced genome. ORF1a (nt 569–5491) encoded a protein of 1640 aa (184K), which contained the MTR (pfam01660) and HEL (pfam01443) motifs at aa positions 81–395 and 1347–1607, respectively, and was apparently the viral replicase. Another alkylated DNA repair dioxygenase (AlkB) motif was identified in ORF1a at aa positions 923–1041. ORF1b (nt 5475–7022) encoded the Closteroviridae_RdRp (cd23253). RdRp is expressed as the C-terminal part of a 241K polypeptide, which is sporadically synthesized as a result of a +1 frameshifting in ORF1a due to ribosomal slippage at nt position 5442. This is the typical mechanism of the RdRp expression in closteroviruses [30].

ORF2 and ORF7 coded for the 55K CPm (p55) of 485 aa and the 39K CP (p39) of 359 aa, respectively. Closter_coat motifs (pfam01785) were revealed in the p55 and p39 proteins at aa positions 301–463 and 176–341, respectively. ORF3 encoded the hydrophobic 6.5K p6 protein of 57 aa. ORF4 encoded the 60K protein (p60) of 544 aa, in which the molecular chaperone DnaK (HSP70) motif (COG0443) was identified at positions 3–478. ORF5 encoded the 53K protein (p53) of 460 aa, in which a viral heat shock protein HSP90 homolog motif (pfam03225) was identified at positions 91–453. ORFs encoding CPm, p6, HSP70, HSP90, and CP can form a quintuple gene transport module, which mediates cell-to-cell transport of the virus genome through plasmodesmata. ORF6 and ORF8 encoded the p22 and p26 proteins, respectively. Negative-sense ORF0 (nt 487–35), potentially encoding a p18 protein 150 aa in size, was found at the 5′ end of genomic RNA. The LChV-2 genome sequence was deposited in GenBank under accession number PZ374890. This is the first report of LChV-2 from Russia.

**Figure 7:**
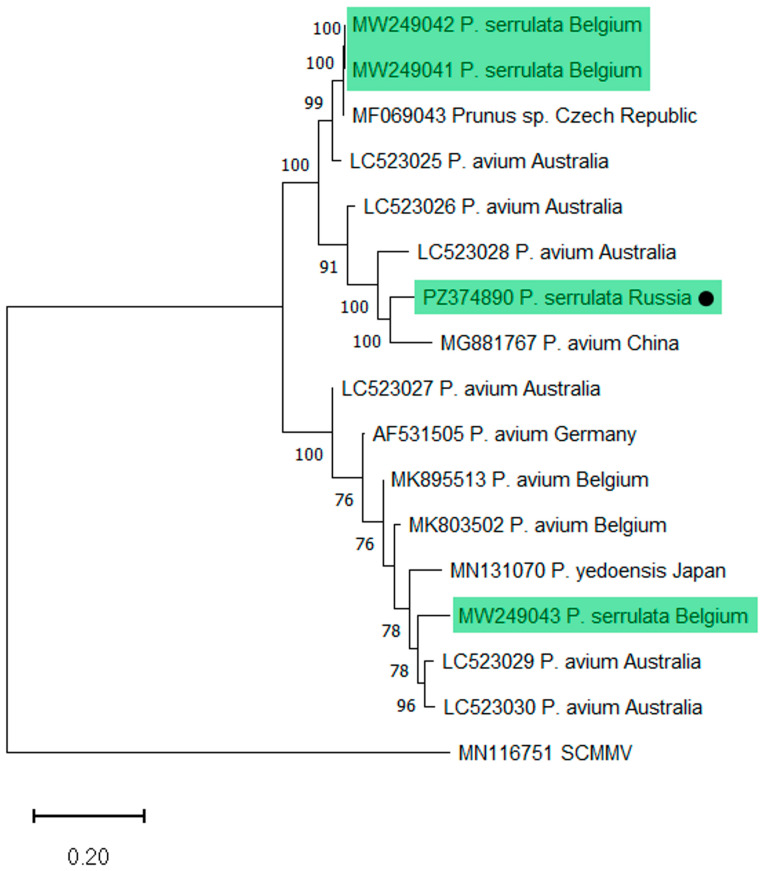
Maximum likelihood phylogenetic trees based on full-length sequences of little cherry virus-2 (LChV-2) genomes using the GTR best-fit substitution model. The accession number, Latin name of the host plant, and geographical origin of the virus isolate are given at the end of the branches. Bootstrap values from 1000 replicates (>75%) are shown next to the corresponding node. The scale bar indicates the number of nucleotide substitutions per site. LChV-2 isolates from *P. serrulata* are in light green, and the Russian LChV-2 isolate is marked with a black circle (•). Sugarcane mild mosaic virus (SCMMV) from ampelovirus subgroup II was used as a phylogenetic outgroup.

The LChV-2-S14 isolate was most closely related (over 89% identity) to the Australian TAS16 (LC523026) and LV27 (LC523028) isolates as well as the Chinese LChV-2-TA isolate (MG881767) from *P. avium* [31,32]. These four isolates formed a common clade in the phylogenetic tree reconstructed from complete LChV-2 genome sequences available in GenBank (Figure 7). In contrast, the LChV-2-S14 shared only 78.0–81.6% identity with the Belgian LChV-2 isolates from *P. serrulata* (MW249041-MW249043) [17,33] and clustered separately from them.

#### 2.2.5. Cherry Virus A

Three distinct CVA-related contigs were assembled in the S14 sample and were named CVA-S14-84, CVA-S14-85, and CVA-S14-86. One more CVA-related contig was assembled in the S27 sample and was named CVA-S27. These contigs were 7389–7432 nt in length, had a poly(A) tail at the 3′ end, and appeared to correspond to complete CVA genome sequences.

Two ORFs typical of this virus have been found in each of these contigs. ORF1 was of 7029 nt and encoded a 266K protein 2342 aa long. The MTR (pfam01660), HEL (pfam01443), Betaflexiviridae_RdRp (cd23245), and Trichovirus_coat (pfam05892) motifs were identified in this polyprotein at positions 43–351, 818–1096, 1283–1600, and 2178–2335, respectively. ORF2 of 1392 nt was completely nested within ORF1 and encoded a 52K protein 463 aa long, which corresponded to the capillovirus MP. ORF1 of the CVA-S14-86 isolate was six nt longer than the rest due to two triplet insertions at positions 1793–1795 and 1848–1850, so the motif boundaries indicated above were slightly displaced in the polyprotein of this isolate. ORF1 was flanked by the 5′ and 3′ UTRs, which were 54–107 and 296–306 nt in length, respectively. The UTR length varied among the novel isolates, apparently because they had not been verified using RACE. The 5′ UTR contained a conserved CTTTACAGAGTCCAAGCTGTAAAG sequence of 57 nt upstream of the ORF1 ATG codon. This pyrimidine-rich sequence can form a stem–loop structure and enhance the translation activity of the CVA genomic RNA through its interaction with 18S rRNA [34].

Three CVA-related genomic RNAs from the S14 sample shared 80.8–83.5% nt identity. Their ORFs 1 were 81.3–83.1% and 85.4–87.1% identical at the nt and aa levels, respectively. ORFs 2 were 90.5–91.5% and 89.2–92.4% identical at the nt and aa levels, respectively. These results suggested that three different CVA isolates were apparently present in the S14 sample. At the same time, the CVA-S27 genome was closely related to that of the CVA-S14-85 isolate, sharing 98.5%, 98.5%, and 99.2% nt identity at the genomic RNA, ORF1, and ORF2 levels, respectively.

RT-PCR with isolate-specific primers was performed to confirm this suggestion. The alignment of four complete CVA genomes revealed several regions with the greatest variability. One of them was a region within the replicase gene approximately 500 nt long. Primers flanking this genomic segment were designed for each of the isolates (Figure S1). As the CVA-S14-85 and CVA-S27 genomes were very similar, the same primer pair was used for their amplification. Four amplicons generated by RT-PCR (Figure 3) were bidirectionally sequenced. The results of the comparison between the complete and partial genome sequences, determined using HTS and the Sanger method, respectively, are presented in Table 2. The partial sequences were 100% identical to the corresponding region of the full-length genome of the same isolate and only distantly related (62.9–69.6% identity) to three other CVA isolates. The only exception was the close CVA-S14-85 and CVA-S27 isolates, which shared 98.2–98.5% identity. Apparently, three distinct CVA isolates are present in the S14 sample. The complete genome sequences of the Russian CVA isolates were deposited in GenBank under accession numbers PZ316344 (CVA-S14-84), PZ316345 (CVA-S14-85), PZ316346 (CVA-S14-86), and PZ316347 (CVA-S27).

NCBI BLASTn showed that the CVA-S14-84 was the closest (98.9% identity) to the Canadian isolates from *P. serrulata* (KY510847) and *P. avium* (KY510879, KY510889) [13]. The CVA-S14-85 and CVA-S27 isolates were most closely related (99.2% identity) to those from *P. serrulata* (KY510861), *P. avium* (KY510856, KY510860), and *P. armeniaca* (KY510876) from another group of the Canadian CVA isolates. The CVA-S14-86 was the closest (82.6% identity) to the Canadian isolates from *P. serrulata* (KY510864, KY510862) and the Belgian one from *P. avium* (MK847263) [13,17].

**Figure 8:**
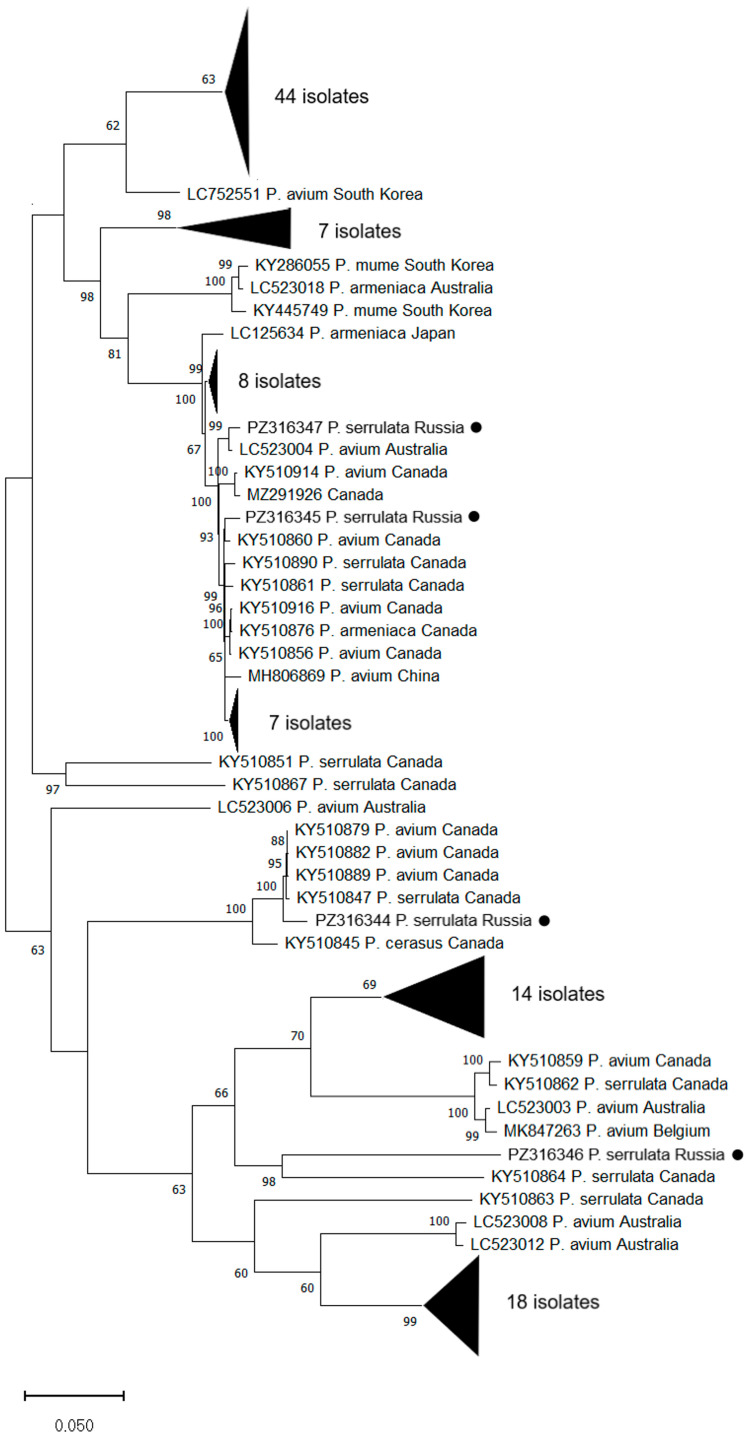
Maximum likelihood phylogenetic tree based on full-length sequences of cherry virus A (CVA) genomes using the GTR best-fit substitution model. The accession number, Latin name of the host plant, and geographic origin of the virus isolate are given at the end of the branches. Russian CVA isolates from P. serrulata are marked with a black circle (•). The black triangles mean the condensed clades. Bootstrap values from 1000 replicates (>60%) are shown next to the corresponding node. The scale bar indicates the number of nucleotide substitutions per site.

Phylogenetic analysis of the complete CVA genome sequences available in GenBank (n = 131) showed that four Russian isolates from *P. serrulata,* in agreement with their nt sequence identity values, were assigned to different clusters, along with their closest relatives (Figure 8).

#### 2.2.6. Recombination in the Genomes of Flowering Cherry Viruses

Several recombination events (REs) were found in the PDV, CVA, and LChV-2 genomes (Table 3). Each of them was predicted by all seven methods implemented in the RDP4 program (RDP, Geneconv, Bootscan, MaxChi, Chimaera, SiScan, and 3Seq) and supported with significant *p*-values.

RNA2 of the PDV-S27 isolate appears to be a recombinant molecule. The recombinant sequence started just before the ORF2 termination codon and extended to the end of the RNA covering the entire 3′ UTR. The Canadian PDV isolates 13C277 from plum (MZ220877) and 13C259 from cherry (MZ220874) were inferred as major and minor parents, respectively. The PDV-S27 and potential major parent genomes shared 93.6% nt identity. At the same time, the putative minor parent and the corresponding recombinant sequence shared only 77.3% identity, suggesting that a more closely related minor parent has yet to be found. No recombination was detected in RNA1 and RNA3 of the PDV-S27 isolate.

Two REs were detected in the CVA-S14-86 isolate. The RE1 and RE2 spanned the 3′ UTR and the RdRp-encoding region, respectively. The Canadian CVA isolate from apricot (KY510862) and the Australian isolate from sweet cherry (LC523007) were inferred as major parents for REs 1 and 2, respectively. Two potential major parents were thus suggested for the CVA-S14-86 recombinant. The Canadian CVA isolate from *P. cerasus* (KY510845) was inferred as a minor parent for the RE2. However, all three proposed parents were only 82.8–85.3% identical to corresponding genome segments of the CVA-S14-86. It is possible that true CVA-S14-86 ancestors have not been discovered yet. No recombination was detected between the three CVA isolates from the S14 sample.

Two REs were also inferred in the LChV-2-S14 isolate. One of them (RE2) was predicted in ORF5, which encoded the p53 protein. The Australian (LC523028) and Belgian (MK895513) LChV-2 isolates from sweet cherry, which were proposed as the major and minor parents, respectively, shared 94.6% and 96.6% identity with corresponding genome regions of the LChV-2-S14 isolate. The Belgian LChV-2 isolate from *P. serrulata* (MW249041) was inferred as a major parent for the RE1, while the minor parent was unknown. Thus, two major parents were proposed for the LChV-2-S14 recombinant. The LChV-2-S14 and Belgian isolate from *P. serrulata* genomes were only 82.0% identical. Due to the higher level of identity (94.6%), the Australian CVA isolate from sweet cherry (LC523028) looks like a more suitable candidate to be considered as a major parent for the LChV-2-S14 recombinant.

## 3. Discussion

This study is the first report on plant virus infections along the Black Sea coast of the Caucasus in Russia and molecular analysis of the detected viruses. Five viruses (PDV, APLPV, AMV, LChV-2, and CVA) were found on ornamental cherry *P. serrulata* trees using HTS and RT-PCR. The S14 tree was shown to be co-infected with LChV-2, APLPV, and CVA of three distinct genotypes, while the S27 tree was co-infected with CVA, PDV, and AMV. Nearly complete genomes of the unveiled viruses were sequenced and characterized. All these showed a genome organization typical of their taxon. This research highlights the value of HTS as a powerful tool that can detect all viruses in a sample and enable assembly of virus genomes from the generated reads.

Over 55 viruses infecting *Prunus* species have been discovered so far, and mixed infections are common. In recent years, the number of known viruses has increased rapidly due to the wide use of HTS for analyzing the viromes of stone fruit trees [31,35,36,37]. AMV occurs worldwide and is one of the most common and well-studied plant viruses, which has been found to infect naturally about 305 plant species, representing 22 families [23]. However, no AMV has been reported on stone fruit crops to date, and no AMV genome sequences from *Prunus* spp. were deposited in GenBank. In this study, AMV was detected on the new host—*P. serrulata* cv. Kanzan. To the best of our knowledge, this is the first report on AMV infection in *P. serrulata* and *Prunus* species in general. Thus, the list of known *Prunus* viruses has been expanded by one, and the AMV host range has also been further expanded.

PDV is widespread in cherry, including in Russia [8,38]. In particular, PDV was found in *P. serrulata* cvs. Kanzan and Amanogawa in mixed infection with other viruses, most frequently with CVA [4,7,13]. The PDV and CVA co-infection has been shown to result in the development of necrotic lesions on Kanzan leaves [7]. However, the symptoms observed on the S27 tree (Figure 1D) did not resemble necrotic lesions and looked completely different. It cannot be ruled out that AMV, the third virus detected in the S27 tree, could modulate the symptoms of infection.

CVA was first reported on *P. serrulata* in California [7]. Later, many CVA isolates were found on *P. serrulata* trees from the plant virus repository of the Canadian Food Inspection Agency, and their complete genomes were sequenced [13]. Seventeen partial MP sequences from Slovakian CVA isolates from *P. serrulata* were also deposited in GenBank (MF048812, MF048836-MF048843, MF048852-MF048859) (unpublished).

Four more CVA isolates were revealed on Japanese flowering cherry in this study. Three of them were found in the same S14 sample, representing different genotypes. Their nearly complete genomes were 80.8–82.6% identical to each other. For each of the CVA-S14-84, CVA-S14-85, and CVA-S14-86 genomes, BLASTn gave significant matches with different CVA isolates. Phylogenetic analysis assigned them to divergent clusters. PCR products obtained with isolate-specific primers were only distantly related to each other, similar to the full-length genome sequences. Thus, the CVA-related sequences assembled in the S14 sample appeared to correspond to three distinct CVA genomes and were not technical artifacts of the assembly (chimeras). It should be noted that multiple CVA genotypes were shown to be often found in sweet cherry, apricot, and flowering cherry, probably as a result of the numerous graftings of *Prunus* material infected with diverse CVA isolates. Multiple genotypes from the same specimen were frequently more closely related to CVA isolates from other specimens than to each other and occurred in different phylogenetic groups [13,31].

APLPV infection in *P. serrulata* was reported from North America, Albania, Italy, Tunisia, Japan, and Palestine [3,8,9]. In this study, APLPV was first detected in Russia, adding information on its global geographic distribution. In addition, this is the first complete APLPV genome from *P. serrulata*.

The epidemiology of the detected viruses remains to be determined. No vector is known for CVA, APLPV, and PDV [20,21,22,26]. LChV-2 can be inefficiently transmitted by mealybugs in a semi-persistent manner and by dodder [27]. PDV is transmitted from plant to plant through pollen and seed [20,21]. AMV is spread in the field by various aphid species in a non-persistent manner [23]. On the other hand, each of these viruses can be readily transmitted through vegetative propagation or grafting. Own-rooted *P. serrulata* trees are difficult to tolerate in the humid subtropical climate of Sochi due to soil waterlogging in winter. For this reason, the flowering cherries growing in this town, including the S14 and S27 trees, have been grafted onto a local sweet cherry rootstock [39]. Sweet cherry is known to be a natural host for the viruses studied in this research. It is highly likely that the rootstocks and/or scions were initially infected with these viruses, raising concerns about their further spread in the town’s green plantings.

## 4. Materials and Methods

### 4.1. Sampling and Sample Processing

The Japanese flowering cherry trees with virus-like symptoms on their leaves were found in June 2024 in the urban green planting in the town of Sochi, located on the Black Sea coast of the Caucasus (N43.585472, E39.723098). These trees were situated two kilometers apart from each other. Symptomatic leaves were collected, bagged, and delivered to the Virology Department of Lomonosov Moscow State University. Total RNA was isolated from fresh leaves using an RNeasy Plant Mini Kit (Qiagen, Hilden, Germany) following the manufacturer’s instructions and stored at −70 °C until use.

### 4.2. High-Throughput Sequencing

cDNA libraries were prepared from total RNA using a TruSeq Stranded Total RNA Library Prep Plant kit (Illumina, San Diego, CA, USA) according to the manufacturer’s protocol. Sequencing was performed on an Illumina NovaSeq 6000 platform (National Research Center “Kurchatov Institute”, Moscow, Russia) to generate 150 nt paired-end reads. Raw reads were quality-trimmed and filtered using FastQC v.0.11.9 and Trim Galore v.0.6.5 (https://www.bioinformatics.babraham.ac.uk/projects/trim_galore; accessed 23 September 2025) with default parameters. Clean reads were assembled de novo into contigs using metaSPAdes v.3.15 [40]. Putative virus-related contigs were identified by BLASTn and BLASTx searches against the NCBI non-redundant nucleotide (nt) and protein (nr) databases. To assess viral read abundance, the cleaned reads were mapped back to the recovered nearly complete viral genome sequences using BBMap v.39.01 (https://sourceforge.net/projects/bbmap/) (accessed on 5 December 2025) with default settings. The average depth of coverage for each viral genomic segment (RNA1, RNA2, RNA3) was calculated as the mean coverage across the aligned positions using the pileup.sh script included in the BBMap suite.

### 4.3. Reverse Transcription Polymerase Chain Reaction (RT-PCR)

Total RNA was also used for the virus RT-PCR detection. The first cDNA strand was synthesized using random hexamer primers and Moloney murine leukemia virus (MMLV) reverse transcriptase (Evrogen, Moscow, Russia). PCR was performed using proofreading Encyclo DNA polymerase (Evrogen) and virus-specific primers developed in-house based on complete genome sequences of the viruses determined in this study (Table S1). PCR products were analyzed by electrophoresis in 1.5% (*w*/*v*) agarose gel, stained with ethidium bromide and photographed using a MultiDoc-It instrument (Analytik Jena US LLC, Upland, CA, USA). PCR products of expected sizes were purified from agarose gel using a BC022 Cleanup Standard Kit (Evrogen) and directly sequenced by the Sanger method using Evrogen facilities.

### 4.4. Sequence Analyses

For an analysis of assembled virus genomes, full-length genome sequences of other isolates of the relevant viruses were retrieved from GenBank. Multiple alignments of nucleotide sequences were carried out using the ClustalW algorithm. The alignments were used to calculate pairwise nucleotide identities between viral isolates and to conduct the phylogenetic analyses in MEGA X [41]. The GTR best-fit nucleotide substitution model was selected using the jModelTest2 package [42]. Phylogenetic trees were reconstructed using the maximum likelihood method with 1000 bootstrap replications. ORFs in the viral genomes were identified using the NCBI ORF finder program (https://ncbi.nlm.nih.gov/orffinder, accessed 19 January 2026). To search conserved domains in viral proteins, the NCBI Conserved Domain Database (CDD, https://ncbi.nlm.nih.gov/Structure/cdd/wrpsb.cgi, accessed 19 January 2026) was used. The Recombination Detection Program (RDP4.101) was used to search potential REs in the virus genomes using the default settings, except that the options “sequences are linear” and “the list events detected by >5 methods” were chosen [43].

### 4.5. Data Availability

The raw reads generated in the S14 and S27 samples were deposited in the NCBI SRA (https://www.ncbi.nlm.nih.gov/sra/PRJNA1449968). The sequences of virus genomes were deposited in NCBI GenBank under accession numbers PZ274394-PZ274396, PZ316344-PZ316347, and PZ374884-PZ374890.

## Figures and Tables

**Table 1:** Results of high-throughput sequencing of *Prunus serrulata* samples with symptoms of virus disease.

Sample	Number of Reads		Number of Assembled Contigs ^a^		Viruses Detected	Average Depth of Coverage ^b^
	Raw	Clean	Total	Virus-Related		
S14	44,232,230	43,859,588	33,421	167	American plum line pattern virus	118× (RNA1) 193× (RNA2) 1046× (RNA3)
					Little cherry virus 2	136×
					Cherry virus A	31×
S27	125,268,054	124,163,192	46,797	175	Cherry virus A	3826×
					Prune dwarf virus	306× (RNA1) 188× (RNA2) 1127× (RNA3)
					Alfalfa mosaic virus	22× (RNA1) 35× (RNA2) 13× (RNA3)

^a^ over 500 bp; ^b^ of genomic RNA with clean reads.

**Table 2:** Identities (%) of complete and partial genome sequences of Russian cherry virus A (CVA) isolates from flowering cherries.

Complete Genome Sequences ^a^ of CVA Isolates	Partial Genome Sequences ^b^ of CVA Isolates			
	CVA-S14-84	CVA-S14-85	CVA-S14-86	CVA-S27
CVA-S14-84	100	69.6	62.9	69.1
CVA-S14-85		100	67.0	98.2
CVA-S14-86			100	68.3
CVA-S27				100

^a^ determined using high-throughput sequencing. ^b^ determined using Sanger sequencing.

**Table 3:** Recombination events inferred in complete genomes of prune dwarf virus (PDV), cherry virus A (CVA), and little cherry virus 2 (LChV-2) isolates from flowering cherry by the Recombination Detection Program v.4.101.

Virus Isolate	Recombination Event	Genome Region ^a^	Breakpoint Positions	Major Parent ^b^	Minor Parent ^b^	*p* Values ^c^
CVA-S14-86	1	3′ UTR	7189–7476	KY510862	Unknown	1.2 × 10^−40^
	2	RdRp	4834–5495	LC523007	KY510845	3.0 × 10^−9^
PDV-S27	1	3′ UTR of RNA2	2385–2577	MZ220877	MZ220874	2.2 × 10^−9^
LChV-2-S14	1	5′ UTR-MTR	410–1192	MW249041	Unknown	4.8 × 10^−78^
	2	p53	11,498–12,036	LC523028	MK895513	1.4 × 10^−71^

^a^ 3′ UTR—3′ untranslated region; RdRp—RNA-dependent RNA polymerase; 5′ UTR-MTR—5′ untranslated region—methyltransferase; p53—viral heat shock protein HSP90 homolog; ^b^ GenBank accession number; ^c^ multiple comparison-corrected probability of false-positive detection of recombination event.

## Data Availability

The datasets used and/or analyzed during the current study are available from the corresponding author on reasonable request.

## Supplementary Materials

The following supporting information can be downloaded at: https://www.mdpi.com/article/10.3390/ijms27125478/s1.
