# Supplementary material for: Molecular Characterization of Viruses Revealed by Japanese Flowering Cherry (Prunus serrulata) Virome Analysis
Source: Int J Mol Sci. 2026 Jun 17;27(12):5478. doi: 10.3390/ijms27125478 (PMC13299341; doi:10.3390/ijms27125478)
Supplement: Supplementary file 1 [file ijms-27-05478-s001.zip › Table S1.pdf]

Table S1. Virus-specific primers for RT-PCR detection of viruses from *Prunus serrulata* trees

№	Virus[a]	Target RNA/gene[b]	Primer	Sequence, 5'-3'	PCR product, bp	Cycling conditions (denaturation/annealing/ elongation), 35 cycles
1	AMV	RNA3/CP	AMVr3-s27-F<br>AMVr3-s27-R	CATACTGCCACAGACGGGCT<br>TAGATCCCTCAGGCAGAGCGT	404	94°C 30 s/52°C 30 s/ 72°C 30 s
2	APLPV	RNA3/CP	AmPcp-F<br>AmPcp-R	CATGACAGAGTAAATCGCGGA<br>GAGGCAACATCCCCAGCGA	570	94°C 30 s/50°C 30 s/ 72°C 45 s
3	CVA	RdRp	cva84-F<br>cva84-R	GAACATTCAAATTAAGTGCTCTA<br>GTCAGCTGYTGATCAGATAACC	491	94°C 30 s/50°C 30 s/ 72°C 40 s
		RdRp	cva85-F<br>cva85-R	TAAAGATTCAAACAACATGCTCAA<br>AGAAAATAGTGCCGGATTTGGA	494	94°C 30 s/50°C 30 s/ 72°C 40 s
		RdRp	cva86-F<br>cva86-R	GCAAGTTCAAGTAAGTTGTCAGT<br>AAGAGGCTGGAATTCAAGCTC	498	94°C 30 s/50°C 30 s/ 72°C 40 s
4	LChV-2	CP	lch2cp-F<br>lch2cp-R2	GTTAACGCTTGGTAGAGGTCGA<br>CTGAGATGTACTTACTCTCGGT	696	94°C 30 s/52°C 30 s/ 72°C 45 s
5	PDV	RNA3/CP	PDVcpF<br>PDVcpR	GGTGTAACGATTGGTTAACTCACT<br>CTAGGAATACTCATAGTGGAAGCA	794	94°C 30 s/56°C 30 s/ 72°C 50 s

[a]AMV – alfalfa mosaic virus; APLPV – American plum line pattern virus; CVA – cherry virus A; LChV-2 – little cherry virus 2; PDV – prune dwarf virus

[b]RdRp – RNA-dependent RNA polymerase; CP – coat protein
