# Supplementary material for: Molecular Characterization of Viruses Revealed by Japanese Flowering Cherry (Prunus serrulata) Virome Analysis
Source: Int J Mol Sci. 2026 Jun 17;27(12):5478. doi: 10.3390/ijms27125478 (PMC13299341; doi:10.3390/ijms27125478)
Supplement: Supplementary file 1 [file Fig. S1.pdf › Fig. S1.pdf]

```
CVA-S14-86      TGCAAGTTCAAGTAAGTTGTCAGTCTCTCACTTTTTCTAGCTCCTTTGATCCAATTCAAACCAACAGTATCTCTTCAATTGGTGATAATGTTCTAGAGAATATCTCTGATAATTTCTTGA 1488
CVA-S14-84      TGAACATTCAAATTAAGTGCTCTACTCTCAATTTTTCAAGTTCCTTTGAATTGGTTGATGCCAATGACATCTCCAGAGTTGGTGAGTGCATCCTCAAAAATATTTCTAGCGGCCTCTCAA 1500
CVA-S14-85      TAAAGATTCAAACAACATGCTCAATCCTCACTTTTTCGAGCTCATTTGAAATGATTGAGACCAACAGAGTTTCCGCAGCTGGAGGATATATCATTGAAAATCTTTCTGATGGTCTATTAA 1485
CVA-S27         TAAAGATTCAAACAACATGCTCAATCCTCACTTTTTCAAGCTCATTTGAAATGATTGAAACCAACAGAGTTTCTGCAGCTGGAGGACATATCATTGAAAATCTTTCTGATGGTCTATTAA 1447
                *  *  *****   *  **       **** ****** ** ** *****     ** *  ****     * **   *  *** *      *  *  * *** * ***       * *  *

CVA-S14-86      ATGTAATCAATGCCTGCAGAAATTGGGTTACCTTTAAAAGATTTGATGGTGGAGGCTATTCAATGGTTAACAGGAATGGTTTGCTTGTTGATTCAATGAAGCAAAAACCTTTTTATTCCT 1608
CVA-S14-84      ATGCTGTTGAAGCCTGCCGAAACTGGGTGACATCAAAAAGATTCTCTGGAGGGGGGTATTCAATGGTTGGAAGAAATGGTCTTCTTGTGGACTCAATGCATCAAAACACCTCCTTTTCTT 1620
CVA-S14-85      GCACTGTCAAAGCTTGCAAGAATTGGTTAACATCAAAGAGATTTTCTGGTGGGGGTTATTCAATGGTCAGCAGGAATGGTCTGCTTGTTGACTCAATGCATCAGAATACCTCTTTCTCCT 1605
CVA-S27         GCACTGTCAAAGCTTGCAAGAATTGGTTAACATCAAAGAGATTTTCTGGTGGAGGTTATTCAATGGTCAGCAGGAATGGTCTGCTTGTTGACTCAATGCATCAGAATACTTCTTTCTCCT 1567
                      *  * ** ***   ** *** * ** *  ** *****   *** ** ** ***********    ** ****** * ***** ** ****** * ** **  * *  *  ** *

CVA-S14-86      CTGAGGTTTACGTGGACCTCTTCCCCTCAACAGTTAGACCCGCCTTTTACTCAGATTCTGCATTCCCATTTTTAGAATCCTTTGAGCCTAAATGGAATTATCTTTCTGGCTCTTGGGATT 1728
CVA-S14-84      CTGAAGTGTTTGTTGACCTCTTCCCATCAGTTGTTAGAGATGCCTTTTACTCGGATCTCAATTTCAGTGTAGTAGAATCATTTGAGCCCAAATGGGATTACCTTCTTGGCTCTTGGATTT 1740
CVA-S14-85      CTGAAGTCTTTGTTGACCTCTTTCCTTCAACTGTTAGGCCTGCTCTTTATTCAGATTCTAACTTCAGTAAATTGGAATCCTTTGAACCTGAGTGGAATTACTTCTCTGGTTCTTGGATTC 1725
CVA-S27         CTGAAGTTTTTGTTGACCTCTTTCCTTCAACTGTTAGGCCTGCTCTTTATTCAGATTCTAACTTCAGTAAATTGGAATCCTTTGAACCTGAATGGAATTACTTCTCTGGTTCTTGGATTC 1687
                **** ** *   ** ******** ** ***     *****     **  **** ** ***       ***       * ***** ***** ** * *** ****  *   *** ******  *

CVA-S14-86      ATAATTTCAGGCAGTTTAGATTATGCGGTCCACAAATTTCATCCAGTGACACGAGTGAAGATGAGGAAGTCAACAAAATTGCTGAAACTCCTGTTACTAAGGATCAAGAGTCAGACATGA 1848
CVA-S14-84      TCAATCACAAACAACTGAGGTTATGTTATGAGGAGGAACAATCAGATGATGATAATAAGGAAGAT---GTCAATAGAGAATCAAATGATTCATCTTTTGAAGGAGTCAAGTCAATTCAA- 1856
CVA-S14-85      TAAATCACAGGCAGTTAAGGTTGTGCCATGAGCAAGATCATTTACATAATGATGACAAGGAGAAG---GCCAGTGAAACCTCTGAAACTTCAAATTCTGAGGAAGTTGAGTCCAATCCA- 1841
CVA-S27         TAAATCACAGGCAGTTAAGGTTGTGCCATGAGCAAGATCATTTACATAATGATGACAAGGAGAAG---GCCAGTGAAACCTCTGAAACTTCAAATTCTGAGGAAGTTGAGTCCAATCCA- 1803
                  ***  **  **  * ** ** **   *    *        *    * *        * **  *    * **    *    *  *   * *   *  * * *      ****

CVA-S14-86      GCTTGAATTCCAGCCTCTTTTCAGAATCTAAGAAAGAGGTAGGAGAAACTTCAAGAAGAT 1908
CVA-S14-84      --GGCCCAGCTTCTCCTTTTGTACAATCCAGTAATGAGGAAGGTGAAACTTCAAAGCAAC 1914
CVA-S14-85      --AATCCGGCACTATTTTCTGTGAGCTCCAAAAATGAGGAAGGAGAATCCTCTAAGCCAA 1899
CVA-S27         --AATCCGGCACTATTTTCTGTGAGCTCCAAAAATGAGGAAGGAGAATCCTCTAAGCCAA 1861
                         *       * *      ** *  ** ****  *** *** * ** *    *
```

Fig. S1 Alignment of partial genome sequences of four cherry virus A (CVA) isolates from *Prunus serrulata* trees. Alignment was performed using Clustal Omega (ebi.ac.uk/jdispatcher/msa/clustalo). The sequences, corresponding to forward (at the top) and reverse (at the bottom) primers are highlighted in color. CVA isolates names are shown in the column to the left of alignment. The numbers to the right of the alignment indicate the position of the last nucleotide in the sequence.
